# Social Capital and Self-Rated Health: Empirical Evidence from China

**DOI:** 10.3390/ijerph17239108

**Published:** 2020-12-06

**Authors:** Jiafeng Gu, Ruiyu Zhu

**Affiliations:** 1 Institute of Social Survey Study, Peking University, Beijing 100871, China; 2 Antai College of Economics and Management, Shanghai Jiao Tong University, Shanghai 200030, China; zhury14@sjtu.edu.cn

**Keywords:** self-rated health, social capital, gender, mobile phone, multi-level Poisson regression

## Abstract

This study assesses the relationship between social capital and self-reported health (SRH) by comparing different genders and ages. It utilizes data from the 2016 China Family Panel Study data with a sample of 30,657 adult individuals from 25 provincial-level administrative regions in China. This was a cross-sectional study conducted with computer-assisted face-to-face interviews to assess social capital and self-rated health among Chinese adults. A multi-level Poisson regression model is employed to model social capital-related dependent variables using the independent variable of fair/poor health status. In terms of social relations, mobile phone use can improve men’s health. However, this effect is insignificant for women. Moreover, gender and age interact with the relationship between social capital and individual health. The relationship between trust and self-rated health is not significantly different between men and women. The frequency of feeling lonely and the lack of feelings for the community in which they live have a negative impact on self-rated health, but there are no obvious differences in terms of gender. The number of meals per week with family members is negatively correlated with men’s SRH, but there is no correlation with adult women 41 and above. Lack of help from neighbors is negatively correlated with men’s health, but not with that of adult women 40 and below. Being a member of the Chinese Communist Party or a member of the Chinese Communist Youth League is positively correlated with SRH for women 60 and above.

## 1. Introduction

The relationship between social capital and health has been a frequent topic of discussion in Western countries over the past two decades [1]. Social capital refers to the resources to which individuals and groups have access through their social networks [2,3]. Self-rated health (SRH) has been regarded as a valid health-status indicator [4,5,6]. The mechanisms and means of social capital’s impact on SRH are influenced by other factors, such as individual lifestyle [7], social norms [8] and politics and the economy [9]. Research had shown that gender plays an important role in the relationship between social capital and health [10,11], yet the mechanisms behind this are not well understood [12,13].

When studying the relationship between social capital and women’s’ health, most scholars report that social capital improves women’s health. Such findings have come out of Sweden [14], the United States [13], European countries [10] and Iran [15]. However, Ferlander et al. [16] found that social capital can be a mixed blessing for women. Silva et al. [17] found that there are inverse associations between mental health and individual level cognitive social capital. Thuy and Berry [18] also found that those mothers had only modest social capital, but the little they had tended to be correlated with better mental health. Amoah et al. [19] found that social capital obstructs efficient healthcare delivery and hurts female inpatients’ health in Ghana. Social capital has also been shown to damage women’s mental health in China [20]. Women tend to bear the cost of creating and maintaining social capital, while deriving fewer benefits from it than men [21]. Women also tend to maintain social capital by making phone calls, while men tend to maintain social capital by meeting in person [3]. However, little research has explored the divergent ways through which men and women maintain social capital.

Moreover, with the popularity of mobile phones, people often use these devices to keep in touch and maintain social capital [22]. There is a positive association between mobile phone usage and social capital [23,24,25]. Although existing literature has demonstrated that using a mobile can contribute to maintaining social capital, the variable effects of social capital maintained by mobile phone usage on women’s and men’s health have received little attention in the literature. There are gender differences in using mobile phones to maintain social capital, and women appear to use mobile phones to maintain social capital more than men [3]. However, despite the proliferation of mobile phones to keep in touch in modern society, evidence of their variable effects on women’s and men’s health via social capital maintenance is still lacking.

What is the mechanism behind social capital’s effect on health? Previous research does not provide a clear explanation. In addition, most research on the relationship between social capital and health has been conducted in a Western context. In-depth analysis of the mechanism of social capital on health in China has been scant. Research on social capital’s effect on Chinese women’s health has been even rarer. Therefore, conducting this research in China not only has important theoretical value, but also practical significance for improving women’s social capital and improving their health.

Based on national representative data, we explore the gender roles, gendered communication and age effects in the relationship between social capital and self-reported health in China, and how social capital might be promoted to enhance women’s health at different ages. We compare the results from China with those of European countries. This provides new evidence on Western-Oriental social capital. Our study not only elucidates how gender affects health in China, but also contributes to the international comparative literature on gender, health, and social capital.

## 2. Literature Review and Hypothesis Development

Social capital was originally a sociological concept. Its origin can be traced to the beginning of the twentieth century [26]. In the health field, the concept of social capital was discussed by Coleman [27], Putnam and Bowling [28] and Kawachi and Berkman [29]. In the existing studies, the operational definitions are inconsistent. This has led to problems in measuring social capital. For example, measurements in existing research do not reflect the multiple dimensions of social capital [30], and some studies only use two questions to measure social capital, oversimplifying the concept [31]. At present, a standardized definition and measurement of social capital has yet to emerge in the field of health research, and there has also been a lack of measurement tools that conform to all dimensions of social capital.

Most existing literature measures social capital by two metrics: social networks and social cohesion [3,10,20]. Thus, social relations, as it relates to social networks and social cohesion, are an important part of social capital [16]. Moreover, social capital is often divided into structural and cognitive domains [2]. Trust measures the structural domains of social capital and subjective feelings measure the cognitive domain [32,33]. Another dimension of social capital is organizational engagement. This can promote organizational social capital, a resource reflecting the character of social relations to the formal or informal organization [34].

How does social capital affect health? Lin Nan et al. have conducted a series of empirical studies on social capital’s effect on personal physical and mental health [35]. They believe that health is affected by stress and resources [36]. Social resources can buffer stress’s effect on health, thereby reducing the negative effects of negative life events on personal physical and mental health [37]. If the multiple dimensions of social capital are considered, then the effect of social capital on health will also have different paths. Although the definitions and measurements of social capital vary across studies, social relations, trust, subjective feelings and organizational engagement are important components of social capital [13,14,30,38]. Therefore, we can explore the path of social capital’s effects on health from these four perspectives.

Social capital is derived from resources embedded in social networks and is rooted in social relations [35,36]. In contemporary society, mobile phone is a convenient communication tool for maintaining social relationships [23]. Information about health promotion can proliferate through social communication circles via mobile phones, which promotes healthy habits [39]. In order to maintain their social circles, people need to socialize with others and have dinner together. As a result, dinners with family members will be less frequent. In other words, if you always eat at home and do not go out to socialize with others, your social network may shrink. In turn, you will obtain less positive information about health promotion from social relationships, which is adverse to your health. Neighborhood relations are an important part of social relations. Mutual help between neighbors can promote each other’s health [40]. Based on the above analysis of the path through which social capital influences health via social relations, our three hypotheses are:

**Hypothesis** **1.** *Mobile phone use has a positive effect on SRH.*

**Hypothesis** **2.** *The frequency of family meals has a negative effect on SRH.*

**Hypothesis** **3.** *Lack of help from neighbors has a negative effect on SRH.*

Social capital is a collection of resources in a social network, either actual or potential. It cannot exist without social trust [41]. When people believe that most others are selfish, it is easy to be suspicious. This is exhausting, physically and mentally, which is harmful to health [42]. On the contrary, trusting others can increase positive interpersonal interaction, which improves health [31,32,38]. In other words, social capital can provide emotional support to promote individuals’ health through social and psychological processes such as interpersonal trust and mutual respect. Based on the above analysis of the path through which social capital influences health via trust, we propose the following hypothesis:

**Hypothesis** **4.** *Trusting others has a positive effect on SRH.*

Social capital can be divided into two types: structural and cognitive. The former refers to an individual’s social network and various forms of social participation, while the latter refers to an individual’s subjective feelings about trust and reciprocity [7,18,29]. At the individual level, social capital often affects personal health through personal subjective feelings [31]. Those who lack social capital are prone to loneliness. This mental loneliness can impair their health [16]. In addition, social capital also affects individual health through community interaction. Community assistance helps improve health [43]. Based on the above analysis of the path through which social capital influences health via subjective feelings, we propose two hypotheses:

**Hypothesis** **5.** *Feelings of loneliness have a negative effect on SRH.*

**Hypothesis** **6.** *Community attachment has a positive effect on SRH.*

Organizational engagement, defined as a socially contextualized pattern of group belonging, is a form of organizational social capital [44]. Individuals with religious beliefs often participate in religious activities regularly. Communication between religious members can promote positive attitudes and contribute to health [45]. In addition, participating in the activities of political parties can also produce additional social capital, generating opportunities to use social resources to improve health [10]. Based on the above analysis of the path through which social capital influences health via organizational engagement, we propose two hypotheses:

**Hypothesis** **7.** *Religious faith has a positive effect on SRH.*

**Hypothesis** **8.** *Communist party or youth league membership has a positive effect on SRH.*

Social capital’s effect on health has been shown to be a source of gender disparities [11,12,46]. Therefore, this study will also test whether those above hypotheses are equally valid for male and female samples. The focus of this research is social capital’s effect on women’s health. Therefore, the discussion will focus more on whether the aforementioned hypotheses hold for the female sample. Age also mediates the relationship between social capital and health [47,48]. Individuals of different age groups have different types and levels of social capital, and their health also deteriorates with age. Therefore, this study will also examine social capital’s effect on women’s health in different age groups.

## 3. Methods

### 3.1. Data Description

The data used in this study come from the adult database of the 2016 China Family Panel Study (CFPS). Initiated in 2010, The China Family Panel Study is a nationwide, comprehensive longitudinal tracking survey conducted by the China Social Science Research Center (ISSS) at Peking University. It aims to reflect Chinese society and the Chinese economy by tracking data at the individual, family, and community levels to capture changes in population, education and health. The CFPS sample covers the population of 25 provinces/municipalities/autonomous regions in China mainland, with the exception of Xinjiang, Tibet, Qinghai, Inner Mongolia, Ningxia and Hainan. The population of these 25 provinces/municipalities/autonomous regions accounts for 95% of Mainland China’s population. CFPS mainly conducts face-to-face interviews aided by computer-assisted personal interviewing (CAPI). In situations where personal interviews are not feasible, telephone interviews using computer-assisted telephone interviewing (CATI) or Web interviews using computer-assisted Web interviewing (CAWI) are substituted.

### 3.2. Health Status

The self-evaluation of the respondents’ health status is this study’s dependent variable. In the survey, respondents were asked “How do you rate your own health status?” and were given five options: (1) excellent; (2) very good; (3) good; (4) fair and (5) poor. A fair and poor health binary variable was generated based on the health self-assessment: fair and poor values were 1, and the rest were 0.

### 3.3. Social Capital

In the public health field, social networks are defined as the extent of connectedness among groups in a society. It is a measure of the network of respondents, and there are three main variables. Credibility of information is important for connectedness among groups [42] and mobile communications, facilitated by mobility and portability of mobile computing can have a positive impact on individual social capital [49,50,51]. Nearly 90% of all respondents use mobile phones, so a monthly mobile phone bill is a good indicator to measure social interaction by mobile phone [51]. The degree of engagement with strong ties such as family members, and with weak ties such as neighbors, is relative to individual social capital [52]. As a result, the second variable is the number of dinners with family members during the week. Family meals are currently an important institution in Eastern and Western societies, serving as normative icons for collective eating [53]. Growing academic research has related family meals to a variety of health outcomes [54,55]. This is coded as an integer from 0 to 7. The third variable is neighbor help [56]. A binary variable of neighbor help was generated: no neighbor help was 0, and others were 1.

The second aspect of social capital is trust, the fundamental factor of social cohesion [20]. This study used one comprehensive variable to measure respondents’ degree of trust. In the survey, respondents were asked “Do you think most people can be trusted, or its better to be more careful with others?” The possible values were: (1) most people can be trusted, and (2) the more careful you are, the better. A trust binary variable was generated based on the answer of this question: the answer that most people can be trusted was 1, and the other was 0.The variable measures trust in the general sense [38]. Note that trust in the general sense measures not the willingness or ability of the respondent to trust, but the degree of trustworthiness of the surrounding environment, as perceived by the respondent.

Subjective feelings are another measure of social capital [33]. The first variable of subjective feeling is loneliness. Survey respondents were asked “Please indicate the frequency of feeling lonely in the past week according to your actual situation”. Possible values were: (1) almost never (less than one day); (2) sometimes (1–2 days); (3) often (3–4 days), and (4) most of the time (5–7 days). A binary variable of no feeling of loneliness was created where answer (1) was coded as 1 while others were coded as 0. The second variable was the level of feeling towards the community. Survey respondents were asked “Do you feel attachment towards the community you live in?” The possible values were: (1) strong attachment; (2) some attachment; (3) average attachment; (4) not much attachment, and (5) no attachment at all. A binary variable of attachment towards community was created where answer (5) was coded as 0 while others were coded as 1.

Finally, to measure organizational social capital, this study considers whether religious beliefs and Communist Party of China or Chinese Communist Youth League membership factor into social capital. YES was coded as 1 and NO was coded as 0.

### 3.4. Control Variables

The control variables added to the model in this study include age and its squared term, gender, marital status, employment status, and education level. Marital status options are: (1) married; (2) unmarried; (3) cohabitating; (4) divorced; and (5) widowed. Marriage was set as the baseline group. Employment status was categorized as: (1) employed; (2) unemployed; (3) in school; and (4) outside the labor market for other reasons. Employment was set as the baseline group. The educational attainment options were: (1) illiterate/semi-literate; (2) elementary school; (3) junior high school; (4) high school/secondary school/technical school/vocational high; (5) junior college; and (6) 4-year college or above. Illiterate/semi-literate was set as the baseline group. They are all binary variables. Moreover, provincial fixed effects are also included.

### 3.5. Model Selection

Most regression models used in the literature to estimate the prevalence ratio are either Poisson regression or log-binomial regression models [57]. In order to consider the possible differences between provinces, a multi-level Poisson regression model is used in this study [10]. This study uses a multi-level Poisson regression model, mainly for comparison with the results of Sara Pinillos-Franco and Ichiro Kawachi’s research [10]. They used a multi-level Poission regression model to analyze social capital’s effect on health in samples from 17 European countries. To facilitate the comparison between China and Europe, it is necessary to adopt the same research method with the same binary dependent variable.

### 3.6. Statistical Modelling

Listwise deletion to analyze complete cases would have resulted in a loss of 15.4% of the sample. Hence, we performed multiple imputations by creating an imputed dataset with the Markov chain Monte Carlo method in order to address potential bias due to missing data [58]. We assumed randomness in our missing variables. The lowest percentage of missing values was 0.09% for our dependent variable, and the highest was 5.32% on the item of neighbor help.

After multiple imputations of the missing values in the sample (*n* = 30,657), we reported descriptive statistics such as mean value and SD of relative variables in total sample, women’s sample (*n* = 15,352) and men’s sample (*n* = 15,305). Multi-level Poisson regression analysis was conducted to test each hypothesis in the theoretical model using R3.5.2, with *p*-value < 0.05 indicating statistical significance. The significance level was set at α = 0.05, and all tests were two-tailed.

## 4. Results

Table 1 reports the descriptive statistics of the variables. 33.5% of the respondents reported that their health was fair or poor. Among men, 29.3% reported that their health was fair or poor, while the proportion of women was 37.8%. This is consistent with many European countries [59]. The mean age of the sample was 46.092 years old, and the average age of women was slightly higher than that of men. The gender ratio in the sample is essentially equal. In terms of marital status, 76% of the sampled population was married. Men had higher rates of singleness and divorces than women, while women had higher rates of widowhood. A total of 70.5% of the samples were employed, and the male employment ratio was 78.1%, higher than that of women. The ratio of women exiting the labor market is 30.5%; for men, it was 15.8%.

In terms of social relations, the average monthly mobile phone bill is CNY 57.19, and men cost more than women. The average number of weekly dinners with family members was 5.581, and participants were more likely to think they could get help from their neighbors. In terms of trust, 56.3% of the samples believed that most people can be trusted. In terms of subjective feelings, 31.7% of the respondents reported feelings of loneliness; 67.9% of the sample felt attached towards the community.

Table 2 reports the prevalence and its 95% confidence interval estimates. The first column shows the results of the full sample, and the second and third columns are the regression results for individual males and females, respectively. To test the discrepancies between males and females, the interaction terms of social capital variables and the binary variable of males were included in model and the results are shown in column 4 of Table 2. As shown, males reported a 14.3% reduction in the fair and poor health status prevalence when compared to females. In terms of marital status, the unmarried demonstrated better health. In terms of employment, those who were employed were in better health. A higher level of education can also significantly improve health. Those with bachelor’s degrees or above had a reduced prevalence rate of 27.4%, compared to those who were illiterate or semi-literate. At the same time, the effect of education on health improvement was greater for females than males.

In terms of social relations, the average monthly mobile phone bill is associated with better health; It means that mobile phone usage can reduce the fair and poor health prevalence rate by 10.5%. However, this effect is not significant for women. Hypothesis 1 was confirmed only in the male sample, not in the female sample. The number of dinners with family members is inversely related to better health. The number of meals per week with family members is associated with a 1.8% increase in fair and poor health prevalence rate. Hypothesis 2 was thus confirmed. Neighbor help can promote health and hypothesis 3 was thus confirmed. As shown, the decline in trust in the general sense is negatively correlated with health, and this finding is consistent with our expectations [38]. Hypothesis 4 was thus confirmed. In terms of subjective feelings, the frequency of feeling lonely and the lack of attachment towards their community are negatively related to health status. Hypothesis 5 and 6 were thus confirmed. Religious beliefs can promote the health of men, while this relationship is not significant for women. Hypothesis 7 was confirmed only in the male sample, not in female sample. Being a member of the Chinese Communist Party or a member of the Chinese Communist Youth League is positively correlated with health status, and this effect is greater for males than females. Hypothesis 8 was thus confirmed. According to the regression coefficients of the interaction terms of the social capital variables and the male dummy variable, the effects of average monthly mobile phone bill, the attachment towards their community, and religious beliefs on health improvement were significantly greater for males than females.

Social capital’s effect on women’s health is age-specific [60,61]. To investigate the relationship between social capital and women’s health, we divided the sample of women into three groups according to age: women between 16 and 40, women between 41 and 60, and women over 60. Table 3 reports the estimated r prevalence rates for these three groups.

In terms of social relations, the average monthly mobile phone bill is insignificant for adult women of all ages. Moreover, the number of dinners with family members is only inversely correlated with better health among women 40 and younger. It is insignificant when women are 41 or above. In addition, lack of help from neighbors is negatively correlated with health among women aged 41 and older. It is insignificant when women are 40 or younger. Trusting others is positively correlated with better health only among women aged 60 or younger. The frequency of feeling lonely is negatively correlated with women’s health, while the attachment towards one’s community is positively correlated with women’s health. Being a member of the Chinese Communist Party or a member of the Chinese Communist Youth League is positively correlated health when women are 61 and above.

## 5. Discussion

Our research shows that some dimensions of social capital predict SRH differently for women versus men. This echoes the findings of previous research [10]. However, women’s SRH was not significantly affected by using mobile phones, while, for men, using mobile phones decreased their prevalence of fair/poor health by 10.5%. As previously discussed, relational use of mobile phones should have a positive effect on social capital, psychological well-being and health [24]. However, our research shows that this relationship only applies to men. Traditional wisdom believes that women like to use mobile phones to maintain social relations, while men like to maintain social capital through face-to-face communication [3]. Though the social capital maintained by usage of mobile phones can improve men’s health, it has no effect on women’s health at all.

Although mobile phones facilitate social capital maintenance, women derive less health benefits from them than men. It is possible that mobile phone usage might not increase women’s social capital. It is easier for women than men to be individualistic and alienated from society because of mobile phone overuse [60]. Additionally, women tend to incur higher health costs creating and maintaining social capital by mobile communication than do men. Studies have shown that feeling or perceiving cell phone ringers triggers more anxiety and depression in women than in men [24,49]. Mobile phones have been associated with stress and sleep disturbances among women, but not for men [49]. Females send more elaborate text messages than males [61]. Women also engage in long phone calls correlated with emotional and personal matters, while men tend to make short phone calls for specific purposes [49].

Women account for as much as 70% of the world’s impoverished population, and about two thirds of the world’s illiterate population [62]. Women also suffer worse health status outcomes than men along several parameters [10,18]. In response, policy makers have sought to implement interventions that empower women’s social capital, with the view that their health and welfare will benefit. Women are encouraged to use mobile phones to extend social networks associated with mutual support in their own communities in order to improve their health and overall quality of life. However, our research reveals that these interventions are only effective for men, because of gendered communication. The social capital maintained by using mobile phones has no effect on women. Thus, women should not be pushed to socialize with mobile phones under the guise of protecting their health.

The number of meals per week with family members is negatively correlated with men’s SRH. However, this form of social capital only negatively affects the SRH of adult women aged 40 or younger. Traditional wisdom on social capital suggests that women tend to be more family oriented, and this form of social capital has positive effects on women’s health [1,59]. However, this is inaccurate. Today, North American women are responsible for about two thirds of routine household tasks [63]. Chinese women face a similar situation. The heavy burden of housework is not only detrimental to women’s health, but also limits the development of women’s social capital outside the family, and even detracts from their work [64]. This negative effect may be diminished among women 41 or over because women’s work and social status have been relatively stable.

Lack of help from neighbors is negatively correlated with men’s health, but not for adult women 40 and below. Trusting others is positively correlated with better health among men, but only for women ages 60 and below. Feelings of loneliness and lack of attachment towards one’s community are both negatively correlated with men’s and women’s SRH. These findings show that gender and age mediate the relationship between social capital and SRH to a certain extent.

The relation between social capital and SRH may be universal, but many cultures will have variations. To European individuals, trust can improve both men and women’s SRH, while the effect of religious faith on men and women’s SRH is not significant [10]. Similar relations were confirmed by the present study, conducted in China. However, differences also exist. To European individuals, working in a political party improves only men’s SRH [10,59]. Meanwhile, in China, being a member of the Chinese Communist Party, or a member of the Chinese Communist Youth League is positively related to the SRH of men, and women aged 60 or older as well. Like men, Chinese women’s SRH can be improved by social capital from participation in politics. This is not the case in Europe. The emancipation and equality of women was one of the main platforms of the communist government of China when it came to power in 1949. This provided Chinese women with opportunities to accumulate social capital by participating in political activities, thus improving their health.

In the context of Confucian culture, individuals are more inclined to maintain and extend social capital through “strong relationships” (i.e., trust and obligation). In “strong relationships”, social interaction is a human relationship network rather than an information bridge, and the acquisition of health-related information is only a byproduct of human relationships [65]. Individuals in European and American societies tend to emphasize “weak ties” in social networks [66]. With weak ties, individuals acquire more heterogeneous knowledge and information. This benefits health based on a wide range of weak social interaction processes. In increasing social capital, mobile phones are an important way for Western societies to maintain weak social interaction. However, in China, in order to obtain valuable information, face-to-face interaction is often necessary. Confucian culture can influence Chinese women’s social style and make them more conservative in social interaction. Because of these reasons, it is difficult for social capital to influence the health of Chinese women via mobile phones. Moreover, the religious beliefs of the Chinese people are utilitarian and lack a sense of repentance, which contrasts with the religious sentiments of Westerners [67]. Therefore, it is difficult for social capital to influence the health of Chinese women via religious beliefs.

In summary, for women, social capital can have a positive effect on health through the following paths: (1) deriving health benefits through socializing with people outside the family; (2) creating a healthy community atmosphere through the mutual help of neighbors; (3) deriving emotional support from trust; (4) accepting and following healthy behavior norms by reducing loneliness and attaching more on the community; (5) strengthening positive values to maintain good mental health by joining the Communist Party or Youth League. For men, in addition to the aforementioned paths, social capital can also improve health by expediting the diffusion of health information on social networks through the use of mobile phones.

This study presents some limitations. First, data are only from China. Therefore, the relationship between social capital and SRH cannot be tested across Asian countries due to lack of data. Second, though several factors were controlled to identify the association between social capital and SRH, factors such as family members’ SRH, family history of illnesses, or social support were not controlled. Third, because this was a cross-sectional study, the establishment of cause-and-effect relationships between social capital and SRH remains limited. These are also issues that need further research in the future.

## 6. Conclusions

This is a study of the disparities between men’s and women’s SRH with respect to dimensions of social capital. It offers new insights into social capital’s effect on SRH among both men and women. This study used the CFPS (2016) adult database to explore gender differences in the relationship between social capital and health. A common argument in the women’s social capital literature is that using a mobile phone empowers women’s social capital and improves their health. This is because women tend to use mobile phones to maintain social capital more than men [3]. However, this is not the case. Our results show that mobile phone usage reduces the prevalence of fair and poor health status by 10.5% among males. However, this factor was insignificant for females. The number of meals per week with family members is negatively correlated with men’s SRH. However, this form of social capital only negatively affects the SRH of adult women aged 40 or younger. Lack of help from neighbors is negatively correlated with men’s health, but not for adult women 40 and below. Moreover, the results indicate that age mediates the relationship between social capital and individual health. Finally, political party participation improves health for men, but only for women over 60. This is different from Europe.

In summary, these findings on the relationship between social capital and health deviate from previous research in terms of the form of social capital, gender difference, gendered communication and demographic variables such as age that constitute the framework of the studies under different cultural backgrounds. These results have implications for global public health policy on improving women’s health.

## Figures and Tables

**Table 1:** Descriptive statistics.

Variables	Total		Men		Women	
	Mean	SD	Mean	SD	Mean	SD
Fair or Poor Health #	0.335	0.472	0.293	0.455	0.378	0.485
Social Relations						
Mobile Phone Bill	57.185	59.075	67.762	66.965	46.604	47.659
Weekly Dinners with Family Members	5.581	2.527	5.337	2.651	5.824	2.373
Neighbor Help #	0.609	0.488	0.614	0.487	0.604	0.489
Trust						
Trust Others #	0.563	0.496	0.579	0.494	0.548	0.498
Subjective Feelings						
No Feelings of Loneliness #	0.683	0.465	0.698	0.459	0.669	0.471
Attachment Towards Community #	0.679	0.467	0.685	0.464	0.672	0.470
Organizational Engagement						
Has Religious Faith #	0.144	0.351	0.119	0.324	0.168	0.374
Communist Party or Youth League Member #	0.205	0.404	0.234	0.423	0.177	0.382
Household Income (per capita)	21867	62778	22511	65209	21226	60256
Age	46.092	17.647	45.764	17.360	46.419	17.922
Male (ref. Female) #	0.499	0.500	1.000	0.000	0.000	0.000
Marriage Status (ref. Married)						
Unmarried #	0.151	0.358	0.181	0.385	0.121	0.327
Co-habitating #	0.004	0.061	0.004	0.064	0.003	0.057
Divorced #	0.018	0.133	0.023	0.150	0.013	0.113
Widow #	0.067	0.249	0.036	0.185	0.097	0.296
Employment Status (ref. Employed)						
Unemployed #	0.011	0.104	0.011	0.105	0.011	0.103
Exited Labor Market #	0.232	0.422	0.158	0.364	0.305	0.460
Studying #	0.052	0.223	0.050	0.218	0.054	0.227
Level of Education (ref. Illiterate or Semi-illiterate)						
Elementary School #	0.211	0.408	0.230	0.421	0.192	0.394
Junior High #	0.283	0.450	0.316	0.465	0.250	0.433
High School/Secondary School/Technical School/Vocational School #	0.143	0.351	0.160	0.367	0.127	0.333
Junior College #	0.059	0.236	0.062	0.240	0.057	0.232
Bachelor’s Degree or Higher #	0.041	0.199	0.046	0.209	0.037	0.189

Notes: the number of total sample, men sample and women sample is 30,657, 15,305 and 15,352; # indicates binary variables.

**Table 2:** Prevalence of reporting fair and poor health values for the whole sample, and for women and men separately.

Variables	PR (95%CI)	PR (95%CI)	PR (95%CI)	PR (95%CI)
	Total	Men	Women	Total
Constant	0.025 *** (0.018–0.034)	0.020 *** (0.013–0.031)	0.025 *** (0.017–0.039)	0.024 *** (0.017–0.032)
Social Relations				
Mobile Phone Bill	0.892 *** (0.871–0942)	0.895 *** (0.874–0.951)	1.003 (0.999–1.005)	1.000 (0.999–1.000)
Mobile Phone Bill*Male				0.999 *** (0.998–1.000)
Weekly Dinners with Family Members	1.018 *** (1.011–1.026)	1.026 *** (1.015–1.038)	1.012 ** (1.002–1.022)	1.015 *** (1.005–1.025)
Weekly Dinners with Family Members*Male				1.007 (0.993–1.021)
Neighbor Help	0.936 *** (0.906–0.967)	0.918 *** (0.872–0.966)	0.952 ** (0.914–0.992)	0.956 ** (0.918–0.996)
Neighbor Help*Male				0.952 (0.893–1.016)
Trust				
Trust Others	0.882 *** (0.855–0.909)	0.895 *** (0.853–0.939)	0.875 *** (0.841–0.909)	0.872 *** (0.839–0.907)
Trust Others*Male				1.025 (0.964–1.089)
Subjective Feelings				
No Feelings of Loneliness	0.724 *** (0.702–0.747)	0.732 *** (0.696–0.770)	0.725 *** (0.697–0.754)	0.723 *** (0.695–0.752)
No Feelings of Loneliness*Male				1.005 (0.945–1.069)
Attachment Towards Community	0.836 *** (0.810–0.864)	0.808 *** (0.767–0.850)	0.861 *** (0.825–0.897)	0.864 *** (0.829–0.901)
Attachment Towards Community*Male				0.928 ** (0.869–0.991)
Organizational Engagement				
Has Religious Faith	0.992 (0.952–1.035)	0.936 *(0.866–1.010)	1.028 (0.978–1.081)	1.025 (0.975–1.076)
Has Religious Faith*Male				0.913 ** (0.836–0.998)
Communist Party or Youth League Member	0.898 *** (0.854–0.944)	0.866 *** (0.810–0.926)	0.936 * (0.867–1.011)	0.903 *** (0.838–0.974)
Communist Party or Youth League Member*Male				0.989 (0.898–1.088)
Household Income(per capita)	1.000 (1.000–1.000)	1.000 (1.000–1.000)	1.000 (1.000–1.000)	1.000 (1.000–1.000)
Age	1.097 *** (1.089–1.105)	1.104 *** (1.091–1.117)	1.091 *** (1.081–1.102)	1.097 *** (1.089–1.106)
Age^2	0.999 *** (0.999–0.999)	0.999 *** (0.999–0.999)	0.999 *** (0.999–0.999)	0.999 *** (0.999–0.999)
Male	0.857 *** (0.829–0.885)			0.925 (0.828–1.035)
Marriage Status ^a^				
Unmarried	0.887 ** (0.797–0.986)	0.945 (0.832–1.074)	0.788 ** (0.645–0.962)	0.887 ** (0.797–0.986)
Co-habitating	1.337 *** (1.092–1.637)	1.334 * (0.954–1.867)	1.373 ** (1.072–1.757)	1.340 *** (1.095–1.641)
Divorced	1.037 (0.924–1.163)	1.093 (0.941–1.268)	0.952 (0.794–1.141)	1.033 (0.921–1.159)
Widow	0.946 ** (0.897–0.996)	1.046 (0.948–1.153)	0.913 *** (0.858–0.971)	0.947 ** (0.899–0.998)
Employment Status ^b^				
Unemployed	1.182 ** (1.003–1.393)	1.206 (0.945–1.538)	1.161 (0.931–1.448)	1.183 ** (1.004–1.394)
Exited Labor Market	1.287 *** (1.242–1.333)	1.441 *** (1.358–1.531)	1.200 *** (1.148–1.253)	1.286 *** (1.242–1.332)
Studying	1.034 (0.838–1.276)	1.087 (0.803–1.469)	1.032 (0.756–1.410)	1.034 (0.838–1.277)
Education Level ^c^				
Elementary	0.981 (0.943–1.021)	1.009 (0.948–1.075)	0.959 (0.911–1.010)	0.979 (0.941–1.018)
Junior High	0.878 *** (0.840–0.918)	0.913 *** (0.854–0.976)	0.852 *** (0.802–0.905)	0.876 *** (0.838–0.916)
High School/Secondary/Technical/Vocational	0.795 *** (0.748–0.845)	0.845 *** (0.774–0.921)	0.752 *** (0.689–0.821)	0.792 *** (0.746–0.842)
Junior College	0.745 *** (0.667–0.832)	0.804 *** (0.688–0.940)	0.690 *** (0.589–0.807)	0.741 *** (0.664–0.827)
Bachelor’s Degree and Higher	0.726 *** (0.630–0.836)	0.883 (0.735–1.060)	0.571 *** (0.456–0.716)	0.721 *** (0.626–0.831)
Provincial Fixed Effects	Yes	Yes	Yes	Yes
Observations	30,657	15,305	15,352	30,657

Notes: *** *p* < 0.01, ** *p* < 0.05, * *p* < 0.1. ^a^ Control group is married. ^b^ Control group is employed. ^c^ Control group is illiterate or semi-illiterate.

**Table 3:** Prevalence of reporting fair and poor health values for the women sample of different age separately.

Variables	PR (95%CI)	PR (95%CI)	PR (95%CI)
	16–40	41–60	Aged 60 and Older
Constant	0.040 *** (0.006–0.255)	0.002 *** (0.000–0.033)	0.321 (0.025–4.061)
Social Relations			
Mobile Phone Bill	1.002 (0.991–1.004)	1.003 (0.994–1.007)	1.003 (0.995–1.008)
Weekly Dinners with Family Members	1.029 ** (1.003–1.056)	1.006 (0.990–1.021)	1.012 (0.998–1.026)
Neighbor Help	1.002 (0.894–1.122)	0.932 ** (0.877–0.991)	0.942 **(0.888–0.999)
Trust			
Trust Others	0.736 ***(0.658–0.822)	0.871 *** (0.822–0.924)	0.960 (0.908–1.014)
Subjective Feelings			
No Feelings of Loneliness	0.572 *** (0.513–0.637)	0.770 *** (0.727–0.816)	0.771 *** (0.729–0.817)
Attachment Towards Community	0.741 *** (0.662–0.830)	0.863 *** (0.812–0.918)	0.925 ** (0.870–0.985)
Organizational Engagement			
Has Religious Faith	1.005 (0.946–1.119)	1.005 (0.931–1.086)	1.006 (0.940–1.077)
Communist Party or Youth League Member	0.988 (0.842–1.159)	0.971 (0.858–1.099)	0.897 * (0.797–1.011)
Household Income(per capita)	1.000 (1.000–1.000)	1.000 (1.000–1.000)	1.000 (1.000–1.000)
Age	1.043 (0.938–1.159)	1.209 *** (1.092–1.339)	1.013 (0.946–1.085)
Age^2	1.000 (0.998–1.002)	0.998 *** (0.997–0.999)	1.000 (0.999–1.000)
Marriage Status ^a^			
Unmarried	0.747 ** (0.577–0.968)	1.498 ** (1.018–2.205)	0.933 (0.511–1.704)
Co-habitating	1.449 (0.691–3.039)	1.226 (0.864–1.740)	1.511 *** (1.260–1.812)
Divorced	0.748 (0.455–1.229)	1.002 (0.798–1.260)	0.998 (0.700–1.423)
Widow	0.895 (0.414–1.935)	0.839 ** (0.731–0.962)	0.927 ** (0.862–0.997)
Employment Status ^b^			
Unemployed	0.907 (0.599–1.374)	1.200 (0.903–1.595)	1.975 *** (1.717–2.273)
Exited Labor Market	0.965 (0.840–1.108)	1.219 *** (1.140–1.303)	1.316 *** (1.233–1.405)
Studying	1.077 (0.753–1.540)		
Education Level ^c^			
Elementary	1.002 (0.838–1.198)	0.960 (0.891–1.035)	0.896 *** (0.825–0.973)
Junior High	0.792 ** (0.659–0.952)	0.896 *** (0.827–0.970)	0.794 *** (0.705–0.895)
High School/Secondary/Technical/Vocational	0.672 *** (0.534–0.844)	0.761 *** (0.677–0.854)	0.856 * (0.723–1.013)
Junior College	0.617 *** (0.467–0.815)	0.745 ** (0.579–0.957)	0.969 (0.693–1.355)
Bachelor’s Degree and Higher	0.644 *** (0.471–0.881)	0.324 *** (0.170–0.619)	0.702 (0.367–1.341)
Provincial Fixed Effects	Yes	Yes	Yes
Observations	6101	5801	3450

Notes: *** *p* < 0.01, ** *p* < 0.05, * *p* < 0.1. ^a^ Control group is married. ^b^ Control group is employed. ^c^ Control group is illiterate or semi-illiterate.
